# Comparison between continuing midwifery care and standard maternity care in vaginal birth after cesarean

**DOI:** 10.12669/pjms.323.9546

**Published:** 2016

**Authors:** Tieying Zhang, Chunna Liu

**Affiliations:** 1 Tieying Zhang, Chief Nurse, Obstetrical Department, Tianjin 4th Centre Hospital, Tianjin, 300140, China; 2 Chunna Liu, Chief Nurse, Obstetrical Department, Tianjin 4th Centre Hospital, Tianjin, 300140, China

**Keywords:** Vaginal birth after cesarean (VBAC), Continuing midwifery care, Standard maternity care

## Abstract

**Objective:**

To determine whether continuing midwifery care has more benefits than standard maternity care in vaginal birth after cesarean (VBAC).

**Methods:**

This study was conducted on women in labour who had history of previous cesarean section and received vaginal birth in obstetrical department of our hospital from May 2013 to November 2014. The included patients were divided randomly into observation group and control group. The women in labour allocated to the observation group received continuing midwifery care, and those to control group received standard maternity care in all the stages of labour. The duration of labor stage together with the rate of fetal distress, neonatal asphyxia, vaginal birth and postpartum bleeding were compared between the two groups.

**Results:**

Ninety-six participants were included in the current study, forty-eight in each group. The length of labor was significantly longer (p<0.05), the vaginal birth rate was significantly lower (p<0.05) and the postpartum hemorrhage rate was significantly higher (p<0.05) in the control group than the observation group. In addition, the rate of fetal distress and neonatal asphyxia were higher in the control group, but there was no significant difference between the two groups (p>0.05).

**Conclusion:**

The continuing midwifery care has more benefits than the standard maternity care in vaginal birth after cesarean (VBAC).

## INTRODUCTION

Cesarean section, which saves many lives of mothers and babies, is the most common procedure in obstetrical department. In the recent decades, the cesarean section rate is increasing sharply.^1^ It was reported that in 2007 the rate of cesarean section was high up to 43.9%, 39.8% and 35.3% in Mexico, Italy and South Korea respectively, and the rate also reached 31.8% in USA.^1^ Many factors, such as decreased training in instrumental vaginal and vaginal breech births, medico-legal issues, the wide use of electronic fetal heart rate monitoring and maternal request, lead to the increasing rate of cesarean section.^2^ Certainly, most of cesarean section cases are not medically indicated. Nowadays the increasing rate of cesarean section is becoming a global issue in the world.

The procedure can bring benefits for women in labour, but it results in more risks, including more pain, longer and difficult postpartum recovery, higher maternal mortality and morbidity, difficulty in conceiving, as well as high rates of stillbirth and miscarriage in subsequent pregnancies.^1^ In addition to that, it leads to a higher likelihood of cesarean section, and many women in labour who had a previous cesarean section will have a routine cesarean section in the subsequent pregnancies.^3^ However, a repeated cesarean section is correlated with more complications and risks such as an increase in operative trauma, placenta praevia, surgical injury and hysterectomy,^4^ even it can cause death of mother and baby. Some authors advocate that vaginal birth after cesarean (VBAC) is a reasonable and safe option for women with previous cesarean section.^3^ Despite the fact, VBAC still has some risks in some conditions, and when VBAC fails, it may result in hysterorrhexis and hysterectomy. As a result, how to improve the safety of VBAC becomes a problem of focuses in obstetrical department.

The continuing midwifery care has been widely used in obstetrical department these years, which includes providing personal support to women during labor and delivery through one-to-one care at the first and second stages of delivery. It provides a continuous, persuasive, and responsive support for woman during delivery.^5^ Also, it may decrease infection in mother and child, lead to more satisfaction of mother and midwife, and increase breast feeding. Hence, we propose a hypothesis that the continuing midwifery care may increase the success rate of VBAC, when compared to the standard maternal care. However, in fact, little evidence is available on the issues.

Therefore, a prospective study was carried out to determine whether the continuing midwifery care has more benefits than the standard maternity care in VBAC.

## METHODS

This study was conducted on women in labour in obstetrical department of our hospital from May 2013 to November 2014. The inclusion criteria included willingness for participating in the study and vaginal birth, not having indications of abnormal delivery such as multiple pregnancies, high risk pregnancy, placenta or amniotic fluid problems, not having mental diseases or problems in which the mother cannot communicate with others,^5^ and all the participants had history of previous cesarean section. The exclusion criteria included mother’s refusal of receiving continuing midwife care and vaginal delivery. The included patients were divided randomly into observation group and control group. The study was approved by the ethics committee of our hospital and all the participants provided written informed consent at the beginning of the study.

### Intervention

The delivery women allocated to the observation group received continuing midwifery care. The midwife provided care during the antenatal, labor and birth, and postnatal periods according to The National Midwifery Guidelines.^6^ During antenatal period, a midwife communicated with the women in labour face to face, to establish mutual trusting relationship between them. At the same time, the midwife provided related knowledge of delivery, to help the delivery woman better understand the process of delivery and kept her relaxed. A psychological counseling should be carried out for delivery women with anxiety, depression or other negative emotions, to help them overcome the fear for delivery and enhance the confidence. During the labor period and postpartum, the same midwife was present continuously at the bedside of women in labour.

The women in labour allocated to the control group received standard maternity care. Antenatal care was provided by antenatal staff including midwives or obstetricians. Staff in the birth unit provided labour and birth care and midwives in the postnatal ward provided postnatal care, and all these care providers were different people.^6^

### Evaluation of outcomes

The age, gestational weeks, interval from last delivery, the thickness of the uterine scar, length of labor stage, together with the rate of fetal distress, vaginal birth and postpartum bleeding were compared between the two groups.

### Statistics analysis

The statistical analysis was carried out using SPSS 19.0 (SPSS Inc., Chicago, IL, United States). The measurement data including age, gestational weeks, interval from last delivery, the thickness of uterine scar, and duration of labor stage were presented as mean±SD. The difference of measurement data were compared using the Student’s *t*-test. The assessment of categorical variables including the rate of fetal distress, vaginal birth and postpartum bleeding was evaluated using chi-squared test. A *P* value < 0.05 was considered as statistical significance.

## RESULTS

In the study, ninety–six participants were included and divided into observation and control group, forty-eight in each group. At the beginning of the study, the data of age, gestational weeks, interval from last delivery as well as the thickness of the uterine scar in all the participants were recorded. The participants age ranged from 25 to 40 years, the gestational weeks ranged from 37 to 42 weeks, interval from last delivery ranged from two to six years, and the thickness of the uterine scar ranged from 1.1 to 2.5 millimeters. There was no significant difference in the abovementioned data between the two groups (p>0.05).

In terms of the length of labor, vaginal birth rate and the postpartum hemorrhage, the data is listed in Table-I. The length of labor was significantly longer in the control group than observation group (p<0.05), the vaginal birth rate was significantly higher in observation group than the control group (p<0.05), and the postpartum hemorrhage rate was significantly higher in the control group than the observation group (p<0.05).

**Table-I:** The comparison between the two groups.

*Group*	*Number*	*Length of labor*	*Vaginal birth*	*Postpartum hemorrhage*
Observation group	48	10.56±2.01min	42(87.5%)	8(16.7%)
Control group	48	14.18±1.35 min	32(66.7%)	18(37.5%)
P value		P<0.05	P<0.05	P<0.05

In addition, fetal distress occurred in six cases in the control group and two cases in the observation group, the rate of fetal distress was higher in the control group, but there was no significant difference between the two groups (p>0.05). Neonatal asphyxia occurred in 5 cases in the control group and 1 case in the observation group, the rate of neonatal asphyxia was higher in the control group than that in the observation group, but no significance was found (p>0.05).

## DISCUSSION

The benefits of continuing midwifery care has been reported widely by many authors.^5^,^7^,^8^,^9^ We carried out a comparative study between continuing midwifery care and standard maternity care in VBAC. Up to now few studies have been published in this regard.

Some authors advocate that different nursing modes may influence the length of labor. In a clinical study from Sehhati, one hundred laboring women were randomly divided into experimental and control groups, obstetrical cares were provided by one midwife from the beginning of phase of labor till postpartum in the experimental group, whereas in the control group, cares were provided by several midwives and without their continuous presence. The results showed that the lengths of labor were shorter in the experimental group than those in control group.^7^ In the current study, we found a consistent outcome with the study of Sehhati. The mean length of labor in the observation group was 10.56 minutes, but the value was 14.18 minutes in the control group, and we found there was significant difference between the two groups, demonstrating that the continuing midwifery care can decrease the length of labor.

At the same time, we found the vaginal birth rate was significantly higher in observation group than the control group. In a study of 4884 laboring women, Rosenstein concluded that the change from a private practice to a collaborative midwifery-laborist model resulted in a decrease in primary cesarean rates and an increase in VBAC rates.^10^ Moreover, in the current study, the postpartum hemorrhage rate was significantly higher in the control group than the observation group. These can confirm the advantages of continuing midwifery care in VBAC.

In addition, we found the rate of fetal distress and neonatal asphyxia was higher in the control group, but there was no significant difference between the two groups. In our opinion, the lower rate of fetal distress and neonatal asphyxia in the observation group can confirm the better effect of continuing midwifery care in VBAC. No significance may be attributed closely to the sample size. A significant comparison of rate using chi square test usually need a relatively larger sample size. Subsequently, we can conclude from current study that continuing midwifery care has more benefits than the standard maternity care in VBAC.

Although VBAC rate are related to many factors^3^,^11^, such as the structure of the maternity care system, the cooperation between midwives and obstetricians and the sociocultural influence, the continuous presence of midwife in all the stages of labor will promote woman’s body to generate endogenous analgesic or endorphin. The continuation of care by one midwife facilitates to find a relationship of mutual trust between the woman in labour and midwife, and enhance the self-confidence and comfort of the woman going to deliver.^7^ It also result in a positive birth experience.^12^ Consequently, in the current study, the observation group has a higher rate of VBAC, and a better outcomes.

However, the current study has its limitation, which lies in the relatively small sample size. We believe a large sample size may be better in explaining the issues. Despite the limitation, the current study provides a positive conclusion for continuing midwifery care, it will help the obstetricians in selection of nursing mode for VBAC.
